# Developing an educational “hub”: impact of a distance-learning curriculum in a multinational cohort

**DOI:** 10.1186/s12909-024-05193-9

**Published:** 2024-04-12

**Authors:** Meridith L. Balbach, Grantly Neely, Afua Yorke, Evangelina Figueroa-Medina, Jonathan Paly, Rebecca M. Shulman, Claire Dempsey, Adam Shulman, Cesar Della Biancia, William B. Cutrer, Benjamin C. Li

**Affiliations:** 1 Vanderbilt University School of Medicine, Nashville, TN USA; 2 Granite Test Prep, Nashville, TN USA; 3 Department of Radiation Oncology, University of Washington Seattle, Seattle, WA USA; 4 Corporación Oncológica México Americana, Aguascalientes, Mexico; 5 Department of Radiation Oncology, Massachusetts General Hospital, Boston, MA USA; 6 Fox Chase Cancer Center, Philadelphia, PA USA; 7 University of Newcastle Australia, Callaghan, Australia; 8 University of Colorado, Boulder, Colorado USA; 9 Rayos Contra Cancer, Nashville, TN USA; 10 Memorial Sloan Kettering Cancer Center, New York, NY USA; 11 UCSF Department of Radiation Oncology, San Francisco, CA USA

**Keywords:** Continuing medical education, Radiation oncology, Global health, cancer, Disparities

## Abstract

**Purpose:**

To address a gap in radiation oncology education in low- and middle-income countries (LMICs), we sought to evaluate the effectiveness and generalizability of a refined curriculum on intensity modulated radiotherapy (IMRT) offered to existing radiation therapy (RT) clinics across Africa and Latin America (LATAM) at no cost.

**Methods:**

A curriculum was created based on prior needs assessments and adapted for participating medical physicists, radiation oncologists, radiation therapists, and trainees in LMICs. English-speaking and Spanish-speaking teams of volunteer educators delivered 27 hour-long sessions 1–2 times weekly for 4 months using video conferencing to African and LATAM cohorts, respectively. Pre- and post-course multiple-choice examinations were administered to LATAM participants, and pre- and post-course self-confidence (1–5 Likert-scale) and open-ended feedback were collected from all participants.

**Results:**

Twenty-five centers across Africa (13) and LATAM (12) participated, yielding a total of 332 enrolled participants (128 African, 204 LATAM). Sessions were delivered with a mean of 44 (22.5) and 85 (25.4) participants in the African and LATAM programs, respectively. Paired pre and post-course data demonstrated significant (*p* < 0.001) improvement in knowledge from 47.9 to 89.6% and self-confidence across four domains including foundations (+ 1.1), commissioning (+ 1.3), contouring (+ 1.7), and treatment planning (+ 1.0). Attendance was a significant predictor of change in self-confidence in “high attendance” participants only, suggesting a threshold effect. Qualitative data demonstrates that participants look forward to applying their knowledge in the clinical setting.

**Conclusion:**

A specialized radiation oncology curriculum adapted for LMIC audiences was effective for both African and LATAM participants. Participant feedback suggests that the refined IMRT course empowered clinics with knowledge and confidence to help train others. This feasible “Hub and Spokes” approach in which a distance-learning course establishes a hub to be leveraged by spokes (learners) may be generalizable to others aiming to reduce global health care disparities through training efforts.

## Introduction

Radiation therapy (RT) may be the most underutilized tool in the pursuit of reducing global cancer care disparities. Investment in RT equipment and personnel in low- and middle-income countries (LMICs) to address worldwide insufficiencies would not only save lives, but also produce long-term economic benefits [1]. In particular, the advent of intensity-modulated therapy (IMRT), a technique leveraging variable beam intensities to deliver higher doses to target volumes with lower toxicities to surrounding tissues, presents a powerful opportunity to improve treatment outcomes [2]. However, knowledge and education gaps remain well-documented barriers to implementing new radiation oncology technologies with high-quality care [1, 3–5].

Significant training is required for radiation oncology professionals to transition from traditional three-dimensional conformal radiation therapy to IMRT safely and effectively [6]. While educational resources ranging from paid online coursework to virtual simulators exist, far fewer target RT centers in LMICs transitioning to IMRT [7, 8]. Such resources may be inaccessible due to high costs, variable foundational knowledge, and cultural barriers. Furthermore, given the pressure to rapidly implement IMRT (e.g., often within 1–2 years) upon acquisition of technology in most LMICs versus a gradual 20-year period experienced in the United States, the footing is unequal for success.

While RT centers in Africa and Latin America (LATAM) increasingly possess IMRT capability, needs assessments by our group in existing African and LATAM RT centers overwhelmingly demonstrated a self-identified need for education in advanced treatment planning procedures and techniques [3, 4, 9]. Without proper training and sufficient experience enabling radiotherapy professionals to deliver care with knowledge and confidence, the abrupt “upgrade” to IMRT may not benefit patients in these settings and could paradoxically result in lower cure rates and higher toxicities [10–13]. To date, no studied intervention exists to promote the safe and effective transition of a radiotherapy department to IMRT technology in LMICs.

We sought to provide a high-quality, culturally accessible curriculum at no cost to medical physicists, radiation oncologists, radiation therapists, and trainees in African and LATAM RT centers. Invoking the “Hub and Spokes” model, we hypothesized this curriculum may act as a “hub” to arm participating centers and learners with knowledge as “primary spokes” [14]. This study assesses the efficacy of this approach to better understand how it might be applied in additional settings outside of radiation oncology.

## Materials & methods

### Development process

**Fig. 1:**
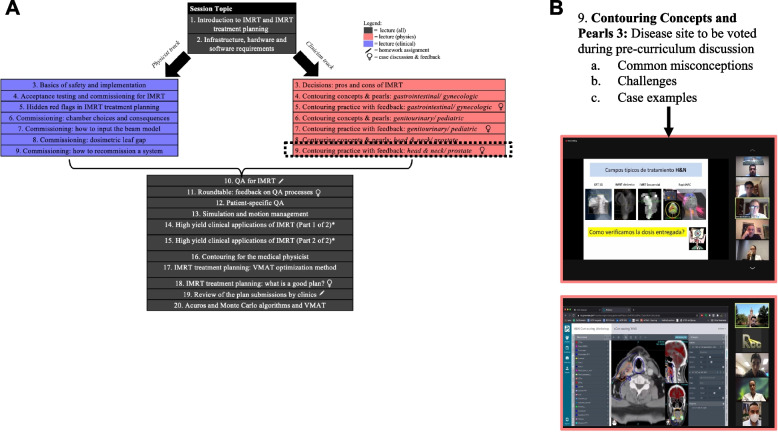
Curriculum schedule and sample clinical contouring unit. **A** The IMRT 2.0 curriculum offered interprofessional sessions applicable to all (dark gray) and allowed participants to select either the physicist (red) or clinician (blue) tracks, yielding an expected total of 20 sessions per participant. **B** Educators were instructed to develop educational materials addressing common misconceptions and challenges with contouring for the selected disease site (in this case, head & neck). Educators delivered introductory interactive presentations with examples. Then, educators and session participants reviewed head & neck contours uploaded by participating centers together, allowing participants to learn from each other

Rayos Contra Cancer (RCC) is a non-profit organization that connects radiotherapy clinics globally to a community of radiation oncology professionals with topic expertise. We performed needs assessments in collaboration with 16 African Core RT leaders and 127 LATAM radiation oncologists as previously described [3, 4]. This led to the identification of limited commissioning, contouring, and quality assurance training for IMRT practices. In response to these needs, we used the Kern six-step approach to curriculum development to design a curriculum [15]. A core curriculum team composed of professionals at large academic centers with over 20 years of IMRT experience, prior experience with curriculum development and teaching at their own institutions, and a strong interest in global health developed a list of fundamental topics for all RT professionals as well as profession-specific topics for medical physicists and radiation oncologists (Fig. 1A). An outline of essential elements for each topic was created to delineate scope.

### Curriculum implementation

To examine the feasibility of our approach, we delivered a pilot IMRT curriculum over 9 sessions via live videoconferencing to 16 medical physicists from 7 cancer centers in Bolivia and Argentina. Participants completed a pre- and post-curriculum self-evaluation, post-curriculum multiple-choice exam, and open-ended feedback. Results were reviewed by the core curriculum team to revise the course syllabus to cover perceived gaps, create a track dedicated to radiation oncologists, eliminate poor exam questions identified via item analysis, and incentivize completion of survey instruments.

Following this pilot, we adapted our curriculum to the unique sociocultural needs of our learners to carry out two programs, one targeting African participants in English and another targeting LATAM participants in Spanish. We delivered 27 hour-long sessions 1–2 times weekly for 4 months using Project ECHO Zoom video conferencing to the African and LATAM cohorts in sequence from Fall 2020 – early 2021 and from early 2021 - Spring 2021, respectively [16]. Content expert educators were identified via inbound requests to volunteer with Rayos Contra Cancer and outbound recruitment of individuals recommended by other educators. Criterion for subsequent selection included prior teaching and global health experience. Using topic outlines, two teams of volunteer educators, one English-speaking and one Spanish-speaking, created instructional materials for their assigned topic, assuming minimal background knowledge (Fig. 1B). English material was developed first by English-speaking educators and then translated and modified into Spanish by Spanish-speaking educators. Interactive didactics and case-based learning utilizing the cloud-based contouring platform ProKnow (ProKnow LLC., Florida, USA) were incorporated to encourage practical participation. Live question and answer (Q&A) was available to all participants via microphone and chat. Asynchronous view or review of recorded sessions was available for all participants, while educators were available for follow-up questions by email. The final curriculum included approximately 23 hours of lecture (with interspersed Q&A), 4 hours of case discussion and feedback, and 5 hours of “homework”.

### Participation

Based on demonstrated interest and apparent need for training, we invited RT centers from 12 clinics across 9 LATAM countries and 12 clinics across Africa and 1 sister institution in Pakistan to participate in the LATAM and African programs, respectively. Each center designated a “clinic coordinator” that worked directly with an assigned RCC representative to disseminate course information and materials. Institution-specific data including staff capacity, patient volume, onsite equipment, delivery techniques, planning systems, and perceived needs was collected via Research Electronic Data Capture (REDCap) [17].

All clinical staff involved in treatment planning or delivery were eligible to participate, with radiation oncologists, radiation oncologists in training, and medical students participating in the “clinician” track while medical physicists, medical physicists in training, dosimetrists, and radiation therapists (who sometimes are involved in treatment planning at African and LATAM centers) participated in the “physics” track. Individual participants were officially enrolled via completion of a pre-curriculum survey. Attendance for each participant was recorded during each session. Participants submitting all pre- and post-curriculum materials, attending > 70% of sessions, and completing 100% of assignments received course certification.

## Evaluation

### Objective measures

Volunteer content expert educators of the LATAM curriculum submitted 1–2 questions pertinent to their assigned topic. Core RCC team members eliminated redundant items to develop a representative 48-item multiple choice test. Pre- and post-course multiple-choice examinations were administered via Google Forms (Google LLC., California, USA) to evaluate change in objective knowledge score [18].

### Subjective measures

Core RCC team members generated 16 items across seven foundations, three commissioning (physics-specific), three contouring (clinician-specific), and five treatment planning (physics-specific) domains. Pre- and post-curriculum surveys were administered via REDCap using a 1–5 point Likert scale across domains to evaluate change in self-confidence. Pre versus post-course changes in each item were averaged to yield four sub-domain and one overall score for each participant. Additionally, mid-course feedback from clinic coordinators and post-course open-ended feedback from all participants was collected. Post-course comments were extracted, iteratively coded, and analyzed for themes [19].

### Statistical analysis

Simple descriptive statistics were used to present data in terms of mean (standard deviation, SD), median (interquartile range, IQR), and proportion (95% confidence interval, CI). Paired sample t-tests and chi-square tests were performed for all available paired quantitative and categorical data, respectively. Pearson correlation coefficient and multivariate linear regression models were used to explore impact of course attendance on outcomes. Spline model regression was used to explore the impact of course attendance for high versus low attendance participants.

All reported *p*-values are two-tailed, with a p-value < 0.05 considered as statistically significant unless otherwise specified. For all data analyses, “Medical physicists” included medical physicists, dosimetrists, and medical physicists in training while “Radiation oncologists” included radiation oncologists and radiation oncologists in training. For all boxplots, whiskers were defined by the maximum and minimum, or in the case of an outlier, an upper and lower fence. Data were processed in a “Google Colaboratory” (Google, Mountain View, CA) environment using Python 3.6.9 (Python Software Foundation, Delaware). Python libraries “Numpy”, “Pandas”, “MatPlotLib”, “StatsModels”, “Plotly”, “Scipy”, and “Kaleido” were imported to extend Python’s native statistical functionality. Python Package “TableOne” was used for the creation of summary tables.

## Results

### Recruitment

We recruited 12 centers in 9 LATAM countries and 13 centers in 6 African countries and 1 sister institution in Pakistan to yield a total of 332 enrolled participants (204 LATAM, 128 African). Documentation of attendees who did not complete the pre-curriculum survey demonstrates that total participation exceeded this number. RT professionals occupying a variety of roles participated. (Table 1).

**Table 1:** Characteristics of 332 individuals and 25 centers participating in the IMRT 2.0 course via Zoom videoconferencing during Fall 2020 – Spring 2021

Characteristic		Count
*Participants*		
**Role**		**n (%)**
	Medical physicist	91 (27.2)
	Radiation oncologist	78 (23.3)
	Radiation oncologist in training	60 (17.9)
	Radiation therapist	50 (14.9)
	Medical physicist in training	26 (7.8)
	Dosimetrist	8 (2.4)
	Medical student	4 (1.2)
	Other	18 (5.4)
**Prior Sources of IMRT Training (non-mutually exclusive)**		**n**
	Informal support of my colleagues	155 (48.1)
	Curriculum in my residency training	129 (38.9)
	Informal support using online resources	94 (29.1)
	Training at a conference or workshop	92 (28.9)
	Other	23 (7.1)
	None	17 (5.3)
**Years of RT Experience, n (%)**		**n (%)**
	< 1 year	18 (5.4)
	1–5 years	134 (40)
	5–10 years	87 (26)
	10+ years	90 (26.9)
*Centers*		**Mean (SD)**
**Course participants**		13.4 (10.6)
**RT staff members**		37.8 (35.7)
**Current use of IMRT**		**n (%)**
	IMRT	11 (44.0)
	No IMRT	14 (56.0)
**Location**		**n (%)**
	Africa	13 (52.0)
	LATAM	12 (48.0)

Diverse sources of prior training were reported with a mean of 6.9 (6.0) years of prior experience. Key characteristics of enrollees completing pre- and post-curriculum surveys, including roles, training status, and mean years of experience did not differ significantly between the LATAM and African programs, suggesting a similar target audience. Both public and private centers participated with a mean enrollment of 13.4 (range 2–40, SD 10.6). Prior to the course, 3 of 13 African (23.1%) and 8 of 12 LATAM (66.7%) centers reported IMRT use, while an additional 7 (53.8%) and 4 (23.3%) anticipated an upcoming transition, respectively.

### Implementation

27 sessions were delivered with a mean of 44 (22.5) and 85 (25.4) participants per session for the African and LATAM programs, respectively. LATAM participants completing pre and post-course materials attended significantly (*p* < 0.001) more sessions than African participants with an average of 18.1 (5.9) and 11.4 (5.1) sessions, respectively. There was no statistically significant difference in the number of sessions attended amongst medical physicists, radiation oncologists, and radiation therapists. An estimated 7740 (6487 LATAM, 1253 African) attendance hours were recorded.

### Objective outcomes: multiple-choice examination

**Fig. 2:**
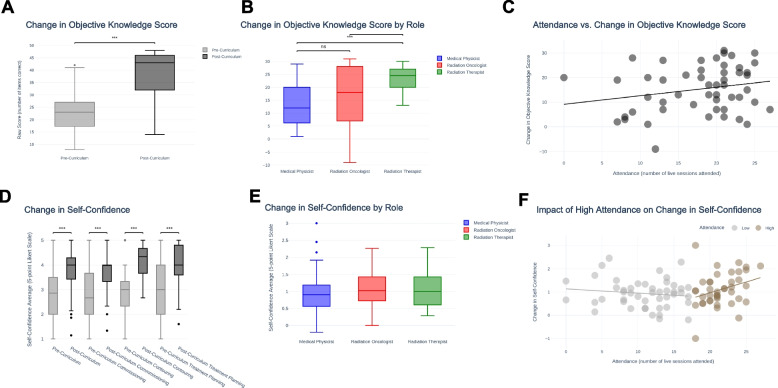
Change in knowledge score via 48-item multiple choice exam (**A**-**C**) and self-confidence via 16-item 5-point Likert scale (**D**-**F**). **A** A paired samples t-test (*n* = 51) revealed significantly improved scores after course completion. **B** There were no significant differences in score change among medical physicists (red), radiation oncologists (blue), and radiation therapists (green). **C** Multivariable linear regression did not identify synchronous attendance as a predictor of score improvement (r^2 = 0.10, F statistic = 2.75, degrees of freedom = 50, *p*-value = 0.07), although a positive linear trend was observed. **D** Paired sample t-tests (*n* = 88) demonstrated significantly improved self-confidence levels across all four domains after course completion (*n* = 88). **E** There were no significant differences in overall self-confidence change among medical physicists (red), radiation oncologists (blue), and radiation therapists (green). **F** Spline regression demonstrated a significant impact of synchronous attendance on change in self-confidence for “high attendance” (defined as attending ≥18 live sessions) participants (r2 = 0.16, F statistic = 6.30, degrees of freedom 36, *p*-value = 0.02)

Participants in the LATAM curriculum were required to complete pre- and post-course multiple-choice examinations to receive certification, yielding 51 paired responses (response rate 25.0%). Course completion correlated with a significant (*p* < 0.001) score improvement, with a median increase from 47.9% (35.4–56.3%) to 89.6% (63.5–95.8%) (Fig. 2A). Furthermore, this significant score improvement held true across participant roles including medical physicists (*p* < 0.001), radiation oncologists (*p* < 0.001), and radiation therapists (*p* < 0.001) (Fig. 2B). Interestingly, there was no significant difference in score change between trainees (including radiation oncologists in training, medical physicists in training, and medical students) and postgraduate learners (including radiation oncologists, medical physicists, dosimetrists and radiation therapists). Furthermore, score increases were similar for participants of centers with current use of IMRT and those without**.**

Multivariable linear regression indicated that attendance non-significantly predicted 10.3% of the variance in score improvement with the following predictive model: Change in Score = .42*Attendance + 6.54*IMRTUse + 2.35 (r^2 = 0.10, F statistic = 2.75, degrees of freedom = 50, *p*-value = 0.07). However, a positive linear trend was observed (Fig. 2C).

### Subjective outcomes: confidence self-evaluation

Participants in the African and LATAM curricula were required to complete pre and post-curriculum self-evaluations, yielding 30 (response rate 23.4%) and 58 (response rate 28.4%) paired responses, respectively. Course completion correlated with significant (*p* < 0.001) median increases in self-confidence across all four domains including foundations (+ 1.1), commissioning (+ 1.3), contouring (+ 1.7), and treatment planning (+ 1.0) (Fig. 2D). This held true across professional roles, with no significant difference in self-confidence change among medical physicists, radiation oncologists, and radiation therapists, suggesting utility of the curriculum for learners of multiple disciplines (Fig. 2E). Both trainees and postgraduate learners displayed similar gains in self-confidence, with no significant difference between groups. Similarly, change in self-confidence did not differ significantly between participants of centers with and without current use of IMRT. LATAM and African participants benefitted alike, with no significant difference in self-confidence change between the two curricula, thus indicating dual efficacy.

Linear regression analysis did not reveal the number of course sessions attended to be a significant predictor of change in self-confidence (r^2 = 0.01, F statistic = .91, degrees of freedom = 84, *p*-value = 0.34**)**. However, considering a cluster of “high attendance, high change” participants, we hypothesized the existence of two distinct learner groups: “high” and “low attendance” participants. In “high attendance” participants, defined as attendance of 18 or more sessions, spline regression analysis demonstrated attendance to be a significant predictor of change in self-confidence (Fig. 2F; r^2^ = 0.16, F statistic = 6.30, degrees of freedom = 36, *p*-value = 0.02). With a final predictive model of Change in Self-Confidence = .11*Attendance - 1.23, attendance accounted for 15.6% of the variance in self-confidence improvement among high-attendance participants. Given the split nature of the curriculum offering only 20 sessions specific to a participant’s track (physics or clinical), these findings indicate that many participants with the greatest gains attended sessions outside of their domain.

### Qualitative outcomes: participant feedback

**Fig. 3:**
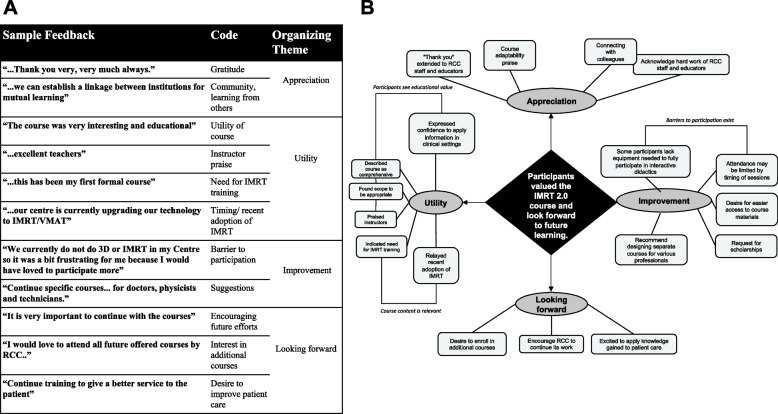
Post-course open-ended feedback suggests utility of the course and future insights. **A** Examples of feedback received with corresponding codes and organizing themes are demonstrated. **B** Following iterative coding and thematic analysis, one global theme (black), four organizing themes (gray), and several basic themes (white) were identified

Participants of both curricula were asked to provide open-ended feedback for the course, yielding 76 unique responses from the African (*n* = 25) and LATAM (*n* = 51) programs. All participant comments were extracted, coded, and analyzed for themes and subthemes as demonstrated in Fig. 3A. Thematic analysis revealed organizing themes of appreciation (*n* = 38), utility (*n* = 40), suggestions for improvement (*n* = 10), and looking forward (*n* = 16) (Fig. 3B). In aggregate, the responses suggest that participants appreciated great value in the IMRT 2.0 course that motivated them to look forward– to how their learning will impact their practice, to seek out further learning, or to suggest opportunities for course improvement.

## Discussion

The IMRT 2.0 curriculum delivered an innovative educational opportunity adapted to learner needs. We identified IMRT as a knowledge gap for participating African and LATAM RT centers, and subsequently leveraged a team of young professionals as central administrators and volunteer professionals interested in supporting global health efforts as described by McLeod et al. to address those needs [5]. Our data support the feasibility and effectiveness of a telehealth-based continuing medical education platform and translatability across African and LATAM regions using shared materials between two English and Spanish-speaking educator teams.

The primary aim of this study was to evaluate the efficacy of this curriculum and identify factors contributing to success (or failure). Objective data assessed with a 48-item multiple choice exam demonstrate significant improvement with course completion. Subjective data in the form of self-confidence evaluations reveal significant increases across all domains. Furthermore, these improvements were seen for professionals occupying a variety of roles with variable prior experience, suggesting utility for all learners.

The number of course sessions attended did not predict change in objective knowledge score or self-confidence overall, although a positive trend was observed for both. However, we did see a significant impact of per-session attendance on self-confidence for those learners that attended at least 18 sessions. These findings suggest that (1) some value of our curriculum is derived from interprofessional learning, as many participants in this cohort attended sessions “outside” of their professional track, and (2) maintaining a high level of learner engagement is crucial to realizing the full potential of this curriculum.

Thematic analysis of course participant feedback may provide a window into how we may achieve greater engagement. While most participant feedback praised the curriculum and encouraged development of future courses like it, suggestions for improvement were prevalent. Chief among these were addressing barriers to participation, including lack of technology to fully participate in sessions, language, and schedule incompatibility. Additionally, patterns of attendance– namely, significantly higher attendance for LATAM versus African participants– may instruct efforts for increasing participation. This finding again emphasizes the value of course offerings in a participant’s first language, given the relatively greater prevalence of native Spanish speakers in LATAM versus native English speakers in Africa. However, it is likely that additional differences between participating African and LATAM centers account for differences in attendance, such as pre-existing relationships or partnerships with RCC or similar organizations, as well as differences in staff availability due to workload, cultural values, and/or internet availability. Future editions of this course may attempt to adjust for some of these barriers by working with center coordinators to ensure technologic compatibility for individual learners, offering coursework in French as requested by several African participants, and offering asynchronous ‘Q&A’ sessions for learners viewing course materials outside of scheduled class.

A key limitation of this study is a limited definition of course engagement. We were not able to track asynchronous views and downloads of course materials, thus preventing us from fully capturing the combined synchronous and asynchronous learning experience. Additionally, some users may have shared screens during sessions, masking attendance data. Qualitative feedback from center coordinators suggests both confounders were at play, thus complicating the association (or lack thereof) between attendance and outcomes. Future studies may use alternative means of measuring engagement to better understand the impact of distance-learning on learner outcomes.

The generalizability of this study may be limited given modest response rates for both subjective and objective data. Our understanding of the curriculum’s impact is restricted to those participants completing course evaluations, inherently selecting for a group that may benefit differently from the intervention than the intended target population in aggregate. Furthermore, this work relies on learner outcomes as a proxy for clinical outcomes, thus evaluating the “reaction” and “learning” levels according to Kirkpatrick’s Four-Level Training Evaluation Model but failing to probe deeper levels demonstrating how learners apply their training [20]. Looking beyond objective knowledge and self-confidence outcomes would enable us to better tailor our curriculum. This might be realized through a study observing changes in treatment practices and patient outcomes following our interventional course.

While other curricula exist for IMRT, this curriculum may serve as a guide for curricula across various disciplines targeting audiences such as ours– that is, practicing professionals of LMICs in need of additional training. We believe that a few key features of our approach enable the success of curricula targeting this group:

1. Our distance-learning curriculum enlisting volunteers and inexpensive cloud-based software makes it possible to deliver education at no cost to participants.
2. Dedication to working with an institution’s needs and cultural preferences (including such obvious factors as language and more obscure ones such as timing) enables development of a faithful partnership.
3. Offering both interactive live and asynchronous learning allows students to take advantage of stimulating learning environments without sacrificing opportunities for those with attendance limited by schedule constraints.
4. Interprofessional learning — with physicists and clinicians learning side-by-side “virtually” — provides a team-based opportunity for professionals to improve their group’s practices together.
5. An emphasis on the role of our participants as the next generation of educators inspires use of their newfound knowledge to transform care as regional leade**rs**.

**Fig. 4:**
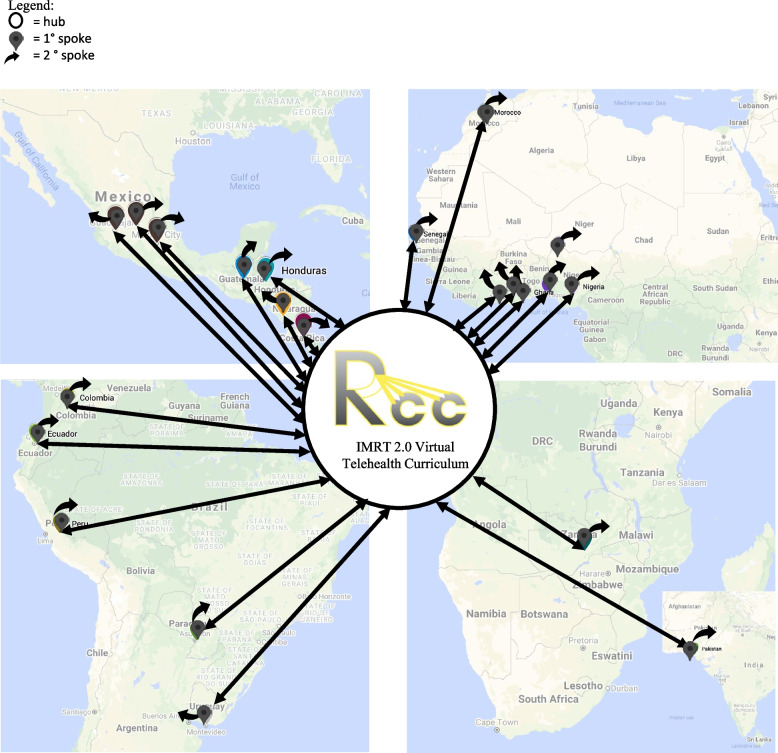
“Hub and spokes” model adapted to LMIC educational efforts. Coordinated efforts by RCC volunteers enabled creation of the IMRT 2.0 curriculum, forming the bulk of the hub. Partnering with 25 centers across Africa and LATAM generated primary spokes. The work invested in creation of the hub will continue to impact patient care as primary spokes act as regional leaders training others

The successful implementation of an educational initiative incorporating these elements has the potential to bring about sustainable impact beyond the participating clinics. However, we suggest this hinges on a curriculum’s ability to achieve #5. Informal feedback from participating centers reveals that course materials travel from our cohort of 332 enrolled participants to colleagues at the same institution and even beyond, supporting our Hub and Spoke model (see Fig. 4). The present study demonstrates that our hub provides an initial foundation through presentations, recordings, and connections with content experts. Critically, however, future studies must examine whether the primary spokes evoke further learning by sharing their learned expertise with others (understood as secondary spokes). Such work would enable us to study whether initial effort expended in creation of the hub sustainably achieves the intended effect– in this case, improvement of cancer care across Africa and LATAM.

## Conclusions

Learner assessment data suggest that the IMRT 2.0 course is serving as an effective “hub”. Further studies are needed to fully characterize the impact of our work, both at the clinical level of “primary spokes” and the didactic level of “secondary spokes”. We suggest that our approach to the hub and spokes model–a distance-learning, culturally adapted live and asynchronous curriculum targeting interprofessional regional leaders– may be useful for others targeting similar audiences in disciplines outside of radiation oncology. Broadened application of such a model has the potential to reduce global disparities in health care.

## Data Availability

The data that support the findings of this study are available from the corresponding author, MB, upon reasonable request at 10.5281/zenodo.7395075 and 10.5281/zenodo.7395083. The educational materials from the refined IMRT curriculum in English and Spanish are available in recorded video format at https://www.rayoscontracancer.org/imrt-for-medical-physicists.

## Abbreviations

RT: Radiation therapy
LMICs: low- and middle-income countries
IMRT: intensity-modulated therapy
LATAM: Latin America
RCC: Rayos Contra Cancer
Q&A: question and answer
REDCap: Research Electronic Data Capture
SD: standard deviation
IQR: interquartile range
CI: confidence interval
